# Supplementary material for: Investigation of CO2 Adsorption on Avocado Stone-Derived Activated Carbon Obtained through NaOH Treatment
Source: Materials (Basel). 2023 Jun 14;16(12):4390. doi: 10.3390/ma16124390 (PMC10304219; doi:10.3390/ma16124390)
Supplement: Supplementary file 1 [file materials-16-04390-s001.zip › materials-2426177-supplementary.pdf]

Supplementary materials

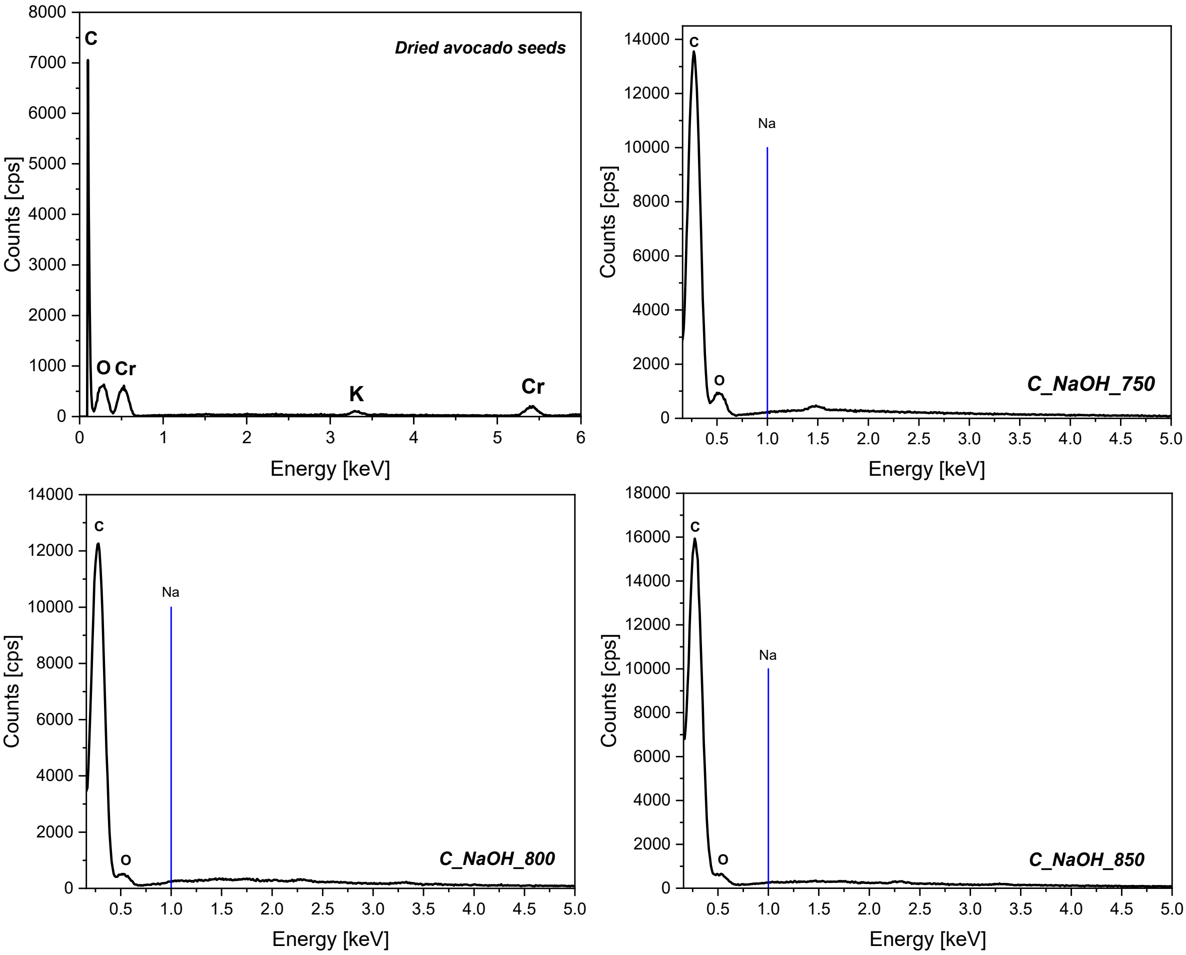

Figure S 1. EDX spectra

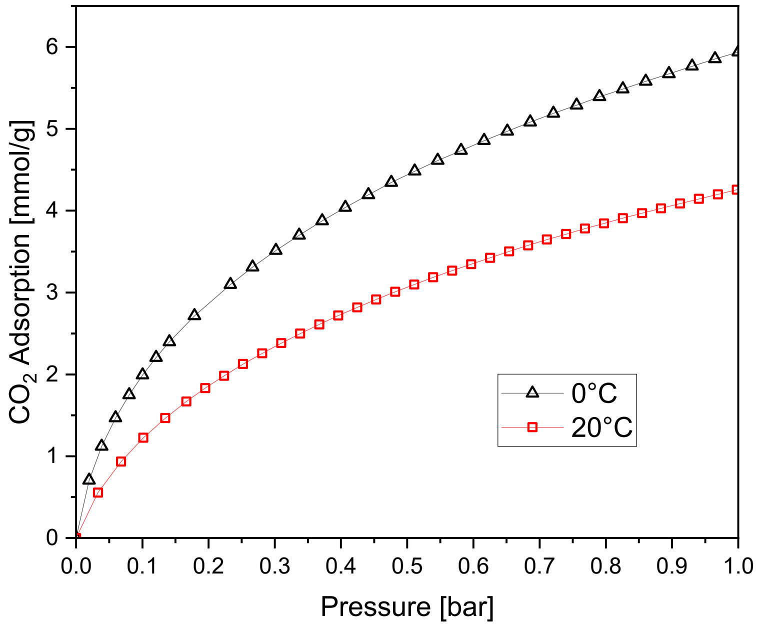

Figure S 2. CO_2 adsorption isotherms at a temperature of 0 °C and 20 °C over C_NaOH_800. The points represent experimental data. The lines were drawn using the Sips model.

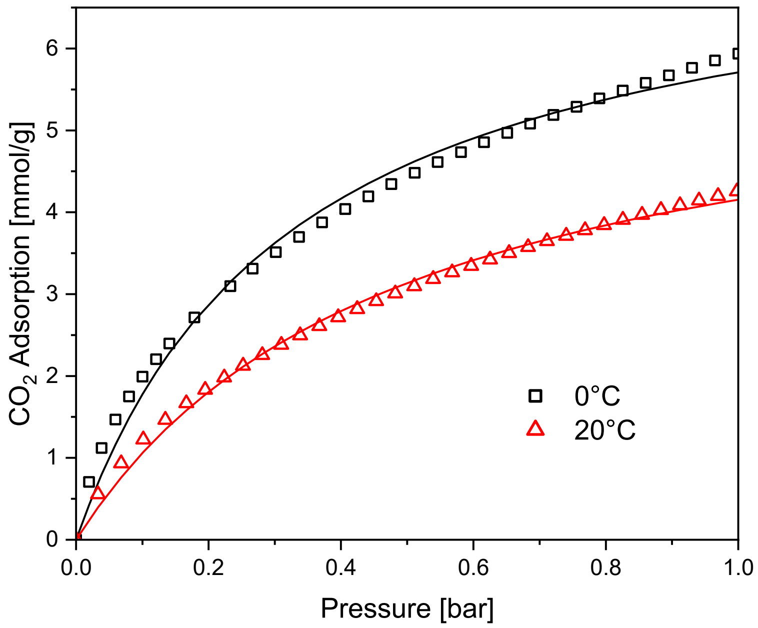

Figure S 3. CO_2 adsorption isotherms at a temperature of 0 °C and 20 °C over C_NaOH_800. The points represent experimental data. The lines were drawn using the Langmuir model.

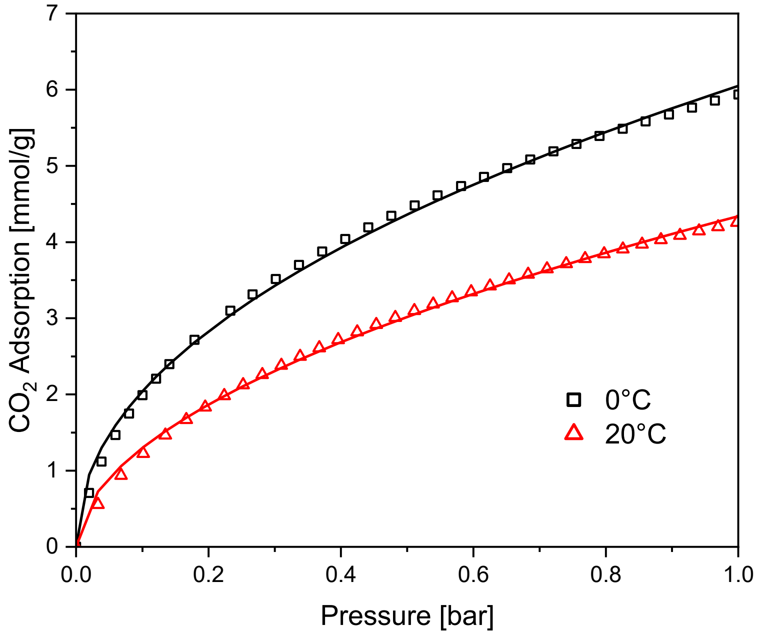

Figure S 4. CO_2 adsorption isotherms at a temperature of 0 °C and 20 °C over C_NaOH_800. The points represent experimental data. The lines were drawn using the Freundlich model.

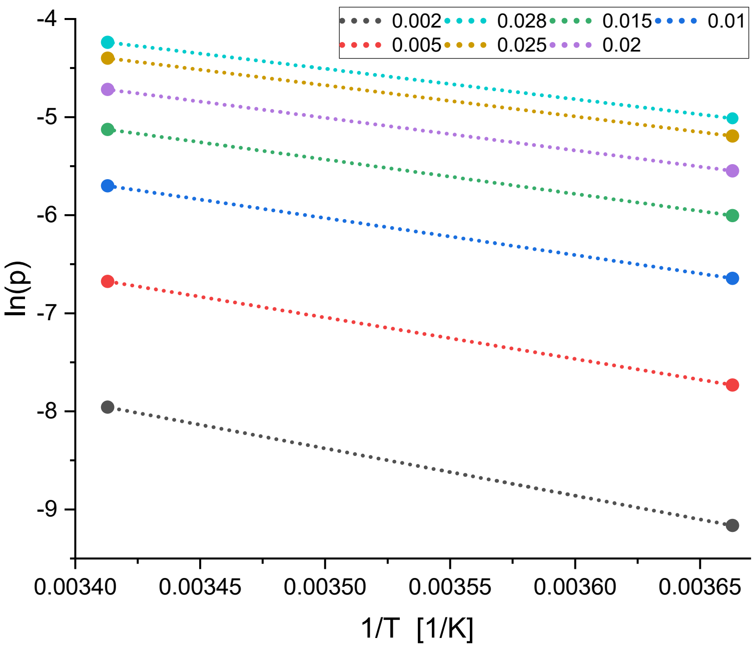

Figure S 5. Adsorption isosteres with different surface coverage

Table S 1. The Langmuir model parameters and standard error calculated based on experimental data of CO_2 for C_NaOH_800

Temperature [°C]	q_m [mmol/g]	b	Error
0°C	7.5756	3.0593	0.7762
20°C	6.1559	2.0760	0.1834

Table S 2. The Freundlich model parameters and standard error calculated based on experimental data of CO_2 for C_NaOH_800

Temperature [°C]	b [bar^{-1}]	n	Error
0°C	6.0480	0.4719	0.2202
20°C	4.3378	0.5240	0.0996

The specific surface area calculation

Specific surface area (SSA) was calculated on the basis of the BET equation in the range of partial pressure of p/p_0=0.05-0.2. This range was narrowed individually for each material so that a linearity of BET plot will be fulfilled. The function:

$$\frac{1}{W\left(\frac{p_0}{p}-1\right)}=\frac{1}{W_mC}+\frac{C-1}{W_mC}\frac{p}{p_0}$$

was plotted. W is the mass of nitrogen adsorbed at a relative pressure p/p_0, W_m is the mass of N_2 constituting a monolayer, p is the partial pressure of nitrogen, p_0 is the saturated vapour pressure of

nitrogen under the temperature of 77 K (N_2 dew point). For microporous materials, the linear region is shifted to lower relative pressures.

W_m was calculated on the basis of slope (s) and the intercept (i) of the linear region of the function.

$$s = \frac{C - 1}{W_m C}$$

$$i = \frac{1}{W_m C}$$

W_m was calculated by combining above equations:

$$W_m = \frac{1}{s + i}$$

The specific surface area was calculated by:

$$SSA = \frac{W_m N A}{M}$$

N is Avogadro's number ($6.023 \cdot 10^{23}$ molecules/mol), M is the molecular weight of N_2 (28.02 g/mol), A is the molecular cross-sectional area N_2 molecule (1.62 nm^2).

The range of linear region was identified by authors, and the SSA was calculated by the software ASAP 2460 version 3.01.

DFT method

The DFT models are created by classical approaches to adsorption as well as models based on modern statistical thermodynamics. There are 14 DFT models in the software ASAP 2460 version 3.01, depending on the pore shape and adsorbent type. The authors chose the best model on the basis of adsorbent properties and goodness of fit vs. regularization. All the calculations were performed in the software ASAP 2460 version 3.01.

Langmuir isotherm model

$$q = \frac{q_{mL} \cdot b_L \cdot p}{1 + b_L \cdot p}$$

where:

q_{mL} – the maximum adsorption capacity

b_L – the Langmuir constant

p – pressure

q – the adsorbed quantity under p pressure

Freundlich isotherm model

$$q = k_F \cdot p^{nF}$$

where:

k_F – the Freundlich constant

n_F – the heterogeneity factor
